# In Silico Exploration of AHR-HIF Pathway Interplay: Implications for Therapeutic Targeting in ccRCC

**DOI:** 10.3390/genes15091167

**Published:** 2024-09-05

**Authors:** Francesco Gregoris, Giovanni Minervini, Silvio C. E. Tosatto

**Affiliations:** Department of Biomedical Sciences, University of Padova, Viale G. Colombo 3, 35121 Padova, Italy; francesco.gregoris@studenti.unipd.it (F.G.); giovanni.minervini@unipd.it (G.M.)

**Keywords:** aryl hydrocarbon receptor (AHR), HIF-1A, VHL, dioxins, ccRCC

## Abstract

The oxygen-sensing pathway is a crucial regulatory circuit that defines cellular conditions and is extensively exploited in cancer development. Pathogenic mutations in the von Hippel–Lindau (VHL) tumour suppressor impair its role as a master regulator of hypoxia-inducible factors (HIFs), leading to constitutive HIF activation and uncontrolled angiogenesis, increasing the risk of developing clear cell renal cell carcinoma (ccRCC). HIF hyperactivation can sequester HIF-1β, preventing the aryl hydrocarbon receptor (AHR) from correctly activating gene expression in response to endogenous and exogenous ligands such as TCDD (dioxins). In this study, we used protein–protein interaction networks and gene expression profiling to characterize the impact of VHL loss on AHR activity. Our findings reveal specific expression patterns of AHR interactors following exposure to 2,3,7,8-tetrachlorodibenzo-p-dioxin (TCDD) and in ccRCC. We identified several AHR interactors significantly associated with poor survival rates in ccRCC patients. Notably, the upregulation of the androgen receptor (AR) and retinoblastoma-associated protein (RB1) by TCDD, coupled with their respective downregulation in ccRCC and association with poor survival rates, suggests novel therapeutic targets. The strategic activation of the AHR via selective AHR modulators (SAhRMs) could stimulate its anticancer activity, specifically targeting RB1 and AR to reduce cell cycle progression and metastasis formation in ccRCC. Our study provides comprehensive insights into the complex interplay between the AHR and HIF pathways in ccRCC pathogenesis, offering novel strategies for targeted therapeutic interventions.

## 1. Introduction

The family of proteins known as basic helix–loop–helix (bHLH) PER-ARNT-SIM (PAS) transcription factors plays crucial roles in physiological adaptations to environmental signals and cancer pathogenesis [1]. Despite having distinct sets of target genes, these proteins form heterodimers by utilizing common dimerization partners within specific subfamilies, leading to intricate interactions [2]. One such protein, hypoxia-inducible factor (HIF), responds to the hypoxic microenvironment by promoting the transcription of its target genes after heterodimerization in the nucleus with the aryl hydrocarbon receptor nuclear translocator (ARNT) [3]. The dysregulation of HIF is linked to key transcriptional programmes in tumorigenesis and directly impacts patient prognosis, particularly in clear cell renal cell carcinoma (ccRCC), the most common form of kidney cancer [3,4]. In ccRCC, the loss of the von Hippel–Lindau tumour suppressor (pVHL) disrupts physiological HIF degradation, yielding constitutive HIF activation [5]. The so-called von Hippel–Lindau (VHL) disease is an inherited condition associated with increased susceptibility to various benign and malignant tumours, including retinal and cerebellar hemangioblastomas, pheochromocytomas, paragangliomas, non-functioning pancreatic neuroendocrine tumours (pNETs), and renal cell carcinoma (RCC) [6,7]. The pVHL acts as a substrate recognition component within a protein complex (VCB), consisting of elongin-B (ELOB), elongin-C (ELOC), and cullin-2 (CUL2) [8,9]. This complex exhibits E3 ubiquitin ligase activity, targeting the HIF-1α transcription factor for ubiquitination and proteasome degradation [10]. Another important protein from the bHLH-PAS family is the aryl hydrocarbon receptor (AHR), encoded by the homonymous gene localizing on chromosome 7, and acting as a 96 kDa ligand-dependent transcription factor [11]. The AHR possesses a bHLH-PAS architecture at the N-terminus and a transactivation domain at the C-terminus, allowing it to dynamically interact with multiple co-activators [11]. When inactive, the AHR forms a complex with stabilizing proteins in the cytosol, including heat shock proteins (HSP90), p23, and XAP2 [12]. Upon ligand binding, the AHR undergoes a conformational change, enabling it to translocate to the nucleus and form a heterodimer with the aryl hydrocarbon receptor nuclear translocator (ARNT). The resulting complex is known to interact with DNA via xenobiotic-responsive elements (XREs) and dioxin-responsive elements (DREs) in gene promoters [13,14]. The AHR responds to diverse ligands, including environmental chemicals, dietary components, and endogenous metabolites. Notably, 2,3,7,8-tetrachlorodibenzo-p-dioxin (TCDD) is a prototype ligand with a planar aromatic structure [15,16]. This compound typically forms as an undesired by-product in the combustion processes of organic materials as well as a secondary product in organic synthesis [17]. In the nucleus, the AHR/ARNT complex transcribes genes involved in detoxification, inflammation, immune response, and development, such as Cyp1a, Cyp1b, GSTA1, EPHX1, and PAI2 [14]. The complex’s transcriptional activity also involves other specific proteins that act as transcriptional modulators, such as p300, CREBBP, NCOA1/2/3, and NRIP1 [18]. The AHR plays diverse roles in cancer, exhibiting both tumour-promoting and tumour-suppressing activities [17]. AHR modulation affects cancer cell behaviour in a cell-specific manner; for example, in breast cancer cells, AHR inhibition enhances proliferation in ER-positive cells but has no effect in ER-negative cells [19]. AHR deletion in various cancer types influences cell proliferation, invasion, and differentiation [20]. Its interaction with signalling pathways like TGF-β, PI3K/AKT/mTOR, NF-κB, FAK/c-Src, and Wnt5a/b-β-catenin further complicates its role [20]. Ligand-activated AHR can inhibit or induce specific signalling pathways, impacting cancer cell functions [19,20,21,22,23,24,25,26]. Moreover, in the last 10 years, the role of the AHR as a therapeutic target in cancers has emerged in particular through the involvement of agonists and inhibitors in breast cancer, hepatocellular carcinoma, and melanoma [20,27,28,29,30,31,32,33]. These complex interactions indicate that the AHR’s function is context-dependent, making it a potential target for cancer-specific therapies, although more cancer-specific studies are needed for a comprehensive understanding. The identified shared transcriptional binding partner, ARNT (HIF-1β), between the AHR and HIF-1α suggests a dynamic interplay and modulation of these canonical pathways. This implies that the preferential activation of one pathway over the other may result in the impairment of specific cellular behaviour and homeostasis. VHL disease recapitulates the persistent competition between HIF1A and the AHR for heterodimerization with ARNT (HIF-1β) and subsequent DNA binding events, which influence the transcription of target genes [34]. By employing a protein–protein interaction network-based approach, we here investigated the impact of pVHL loss on the regulation of AHR activities as well as their intricate pathways interplay [35,36,37]. We identified AHR-specific interactors and used them to predict a set of biological responses that may be compromised by the constitutive activation of HIF-1α [34]. By integrating our analysis with expression data from exposures to TCDD and expression data from pVHL-defective clear cell renal cell carcinoma (ccRCC), we provide insights into the potential repercussions of an impaired AHR pathway in ccRCC and its subsequent impact on tumour progression. Furthermore, the accompanying Kaplan–Meier analysis sheds light on the association between numerous AHR interactors and unfavourable survival rates in ccRCC, collectively supporting the intricate and miscellaneous role of the AHR in the tumour microenvironment. Our data propose the AHR as a potential target for the simultaneous agonistic activation of its regulative function, such as modulating androgen receptor (AR) transcriptional activity and protecting the retinoblastoma-associated protein (RB1) against phosphorylation to reduce cell cycle progression [19,38,39,40,41,42]. These three proteins indeed present low expression levels and are linked to low survival prognoses in ccRCC. In this specific scenario, AHR-selective activation could result in an interesting therapeutic target for treatment against pVHL-null ccRCC.

## 2. Materials and Methods

### 2.1. Network Generation

The protein–protein interaction network data were retrieved from five of the most reliable databases of protein interaction, these being BIOGRID, HIPPIE, STRING, IntAct, and KEGG [43,44,45,46]. In particular, these databases were selected as they store experimentally validated data. To highlight differences in protein network composition upon exposition to TCDD only, AHR direct interactors were included, while interactions between the AHR interactors were filtered and not considered in the final network. We employed Cytoscsape 3.9.1 for network data handling, and the interaction data from databases were merged into a single network [47,48]. Each network was filtered to include only experimentally validated interactions. In the case of the KEGG network, we exploited the KEGGREST package (version 1.42.0 and Bioconductor 3.17) in R (version 4.3) to perform an integrative merge of selected KEGG pathways where the ARNT is involved, specifically the HIF1A signalling pathway (hsa04066), Cushing syndrome (hsa04934), pathways in cancer (hsa05200), chemical carcinogenesis—receptor activation (hsa05207), chemical carcinogenesis—reactive oxygen species (hsa05208), renal cell carcinoma (hsa05211), and Th17 cell differentiation (hsa04659) [49,50]. In detail, databases were searched imposing the following filters:

- STRING: interactions with an “experimental score” > 0 and no text mining;
- BIOGRID: interactions derived from experiments with a “Biogrid_score” > 0;
- KEGG: manually curated interactions from pathways including the ARNT;
- HIPPIE: this database uses a specific scoring system; we selected interactions with a score > 0.5;
- IntAct: interactors defined by the terms “association” and “physical association”.

Functional annotations of AHR interactors were obtained from the manual curation of papers describing the interaction as reported in the different databases. All databases were accessed in August 2023.

### 2.2. NCBI-GEO Expression Profiles

The data for protein levels were retrieved from Gene Expression Omnibus (GEO), a free public database of microarray/gene profiles [51]. In this study, we employed expression profiles that describe the dioxin effect on HepaRG, MCF7, Ishikawa cells, and HepG2. The cells were subjected to 100 nM of TCDD for six hours (GSE69844, GSE69845, GSE69849, and GSE69850) [52]. The second set of expression profiles refers to datasets describing the ccRCC condition (GSE36895, GSE102101, GSE107848, and GSE186013); here, the first two datasets are from patient samples, whereas the last two datasets are from 786-O cell lines. GSE36895: the RNA of clear cell renal cell carcinoma (ccRCC) primary tumours, tumours growing in immunodeficient mice (tumorgrafts), and normal kidney cortices were labelled and hybridized to Affymetrix Human Genome U133 Plus 2.0 arrays [53,54]. GSE102101: RNA-seq profiles of 10 patient-matched normal kidney and ccRCC pairs [55]. GSE107848: the transcriptomic profiles of 786-O under normoxia, short-term hypoxia, and long-term hypoxia were analyzed using next-generation sequencing [56]. GSE186013: transcriptomic profiles of 786-O-TR-Ctrl and 786-O-TR-VHL [57].

### 2.3. DEG Definition

The expression profiles were analyzed using the GEO2R online tool to compare two or more groups of samples to identify genes that are differentially expressed across experimental conditions, and default constraints were applied [51]. Genes with logFC > 0 have been defined as upregulated, and those with logFC < 0 as downregulated [58]. Moreover, to overcome the differences within the datasets, since different cell lines were treated with TCDD and the ccRCC datasets were from both patients and cell lines, proteins were defined as up- or downregulated if they had the same expression levels in at least three of the five datasets; if this was not the case, they were declared as undefined expressed and not considered.

### 2.4. Survival Analysis of DEGs in Renal Cell Carcinoma

Kaplan–Meier plots are commonly used for assessing the effect of a great number of genes on survival based on the EGA, TCGA database, and GEO (Affymetrix microarrays only) [59,60]. The log-rank *p*-values and hazard ratios (HRs) with 95% confidence intervals were computed for ccRCC and shown on the plots for each protein. The expression levels that define the effect on the patient’s survival rate were compared with the levels expressed in the GEO datasets for ccRCC. *p*-value evaluation and correction were performed by applying the Benjamani–Hochberg FDR method [61].

## 3. Results

### 3.1. Description of PPIN Features

**Figure 1:**
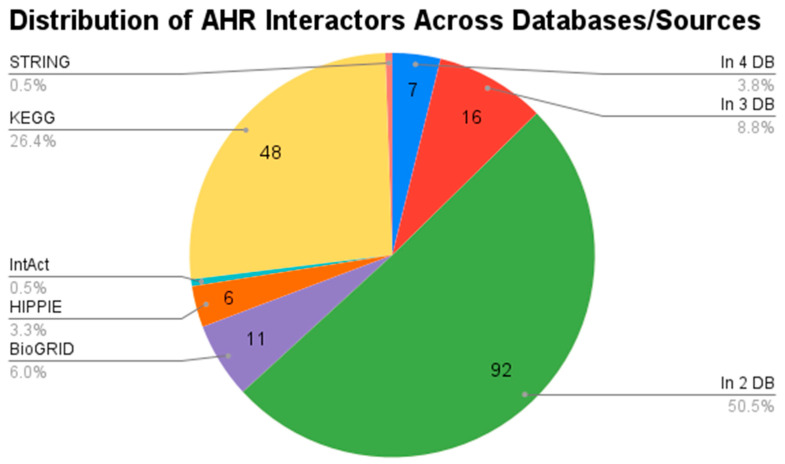
Pie chart showing the distribution of AHR interactors across databases.

All interactions considered for constructing the AHR interactor networks underwent rigorous experimental validation and curation. The merged network, combining data from various databases, encompasses 182 nodes connected by 327 edges. This protein–protein interaction (PPI) network is centred around AHR, offering the most comprehensive view of its interactors. Figure 1 illustrates the sources of interactions in the merged network, with the majority stemming from at least two databases.

Notably, the database with the highest number of unique interactors was KEGG. This underscores the robustness and reliability of our AHR-centred PPI network, providing a thorough understanding of its interacting partners. The specific dimensions of each network are detailed in Table 1.

### 3.2. DEG Network Representation

**Figure 2:**
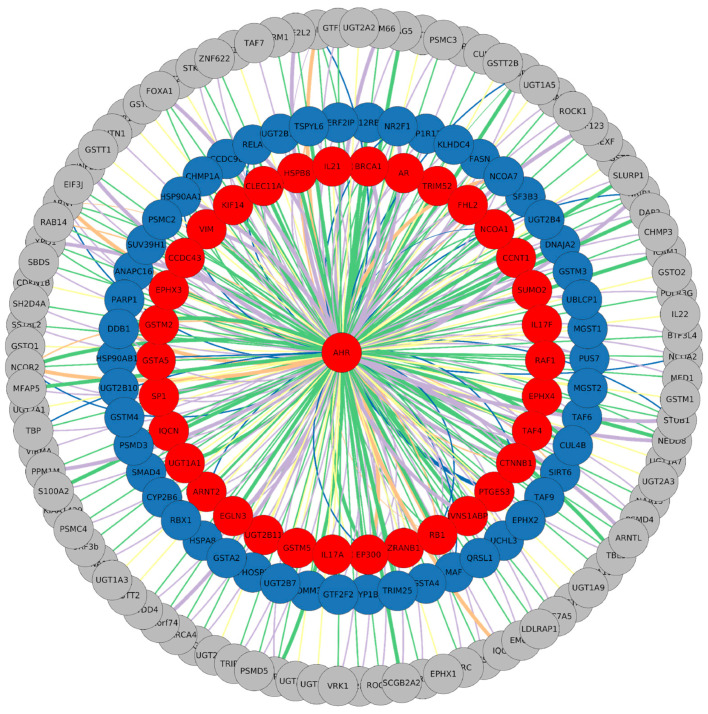
Merged AHR protein–protein interaction network. The colour code represents interactors following 6 h exposure to 100 nM of TCDD. Red is for upregulated proteins and blue is for downregulated, while nodes with an undefined expression level are represented in grey. Edges are coloured according to their node derivation: green is for interactions found in BIOGRID, violet represents data from HIPPIE, and orange for those from IntAct, while yellow and blue are for KEGG and STRING, respectively. The thickness of the lines represents the confidence score of the interactions as defined by each database, while the colours denote the different sources of the interactions, with each database assigned a distinct colour.

The network serves as a crucial tool for visualizing and interpreting the expression levels obtained from the GEO datasets. TCDD-related expression specifically identifies interactors that are differentially expressed following the activation of the canonical AHR pathway. The datasets under consideration involve four distinct cell lines, including HepaRG, MCF7, Ishikawa cells, and HepG2, all subjected to treatment with 100 nM of TCDD for 6 h (GSE69844, GSE69845, GSE69849, and GSE69850). The visualization of these data is shown in Figure 2, where differentially expressed genes (DEGs) are represented in various colours: 35 are upregulated, 50 are downregulated, and the majority exhibit undefined expression across the analyzed datasets.

**Figure 3:**
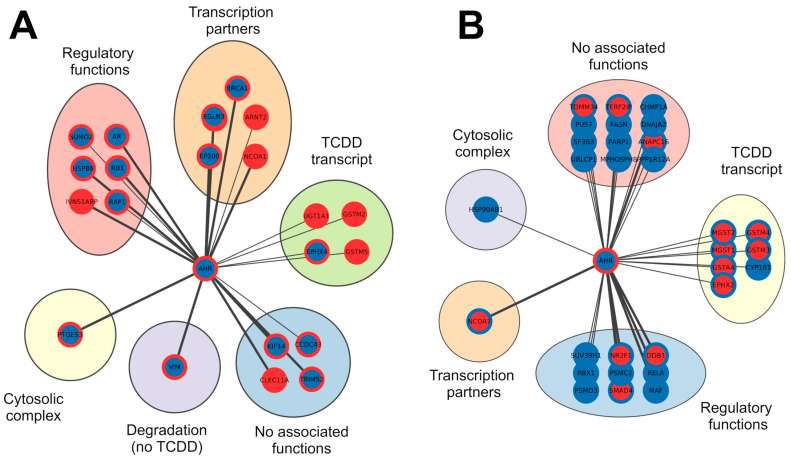
Networks of interactors clustered by functional relationship with the AHR. Panel (**A**) groups proteins upregulated after exposition to TCDD, while downregulated nodes after exposition to TCDD are reported in panel (**B**). Red borders mark upregulated nodes, while blue is for those that are downregulated. Fulfilled red or blue nodes are used to highlight up- or downregulated nodes in both TCDD and ccRCC samples. The thickness of the lines represents the number of sources reporting the interaction.

Through this representation of expression data, we can discern which AHR interactors are the most commonly associated with the activation of its canonical pathway. By mining data from different cell lines, we identified a minimal set of interactors reliably influenced by dioxin. Notably, well-known AHR target genes such as CYP1A and CYP1B are downregulated, suggesting that cell type can influence target gene expression. Conversely, other target genes implicated in xenobiotic metabolic processes, such as glutathione S-transferase A5 (GSTA5), glutathione S-transferase Mu 2 (GSTM2), and UDP-glucuronosyltransferase 2B11 (UGT2B11) [12], were found to be upregulated. This nuanced exploration sheds light on the complex relationship between the AHR and its interactors in response to TCDD across diverse cell lines. We categorized interactors into two main groups, namely upregulated and downregulated, and compared their expression levels during canonical pathway activation with their expression data derived from ccRCC, a well-known scenario characterized by the absence of functional pVHL. The previously selected upregulated and downregulated proteins were integrated with a second set of expression levels referring to the ccRCC condition. Similar to the first dataset, we applied the same colouring code and procedures defining differentially expressed genes (DEGs), resulting in proteins clustered as upregulated, downregulated, or having undefined expression. We found that within the established AHR interactor DEG pool, 35 and 30 proteins were upregulated and downregulated in ccRCC, respectively. Table 2 summarizes the DEGs that were considered for further analysis, presenting the levels of expression in the two conditions. To gain insights into the biological function of these interactions, we annotated each interactor with information derived from the literature. These interactors were grouped into six clusters, namely “Cytosolic complex”, “Regulatory functions”, “Transcription partners”, “TCDD transcript”, “Degradation (no TCDD)”, and “No function”, based on their specific interaction nature/function with the AHR (Figure 3). Moreover, in a broader context, we propose that proteins with high expression levels in both scenarios may not be induced solely by the canonical AHR pathway. Higher expression upon dioxin exposure paired with lower expression in ccRCC is likely dependent on AHR activation, while lower expression in the first dataset and higher expression in ccRCC could indicate that these genes are specifically promoted in the kidney tumour context. Finally, we were unable to discriminate low expression levels in both scenarios as they may be related either to the tumoral environment or attributed to the cell lines selected in TCDD datasets (Table 2). Nevertheless, our interactor categorization should provide a comprehensive understanding of the intricate dynamics of the AHR protein binding network in different scenarios, shedding light on potential biological functions and implications in the context of ccRCC.

### 3.3. Analysis of the DEGs by a Kaplan–Meier Plotter

**Figure 4:**
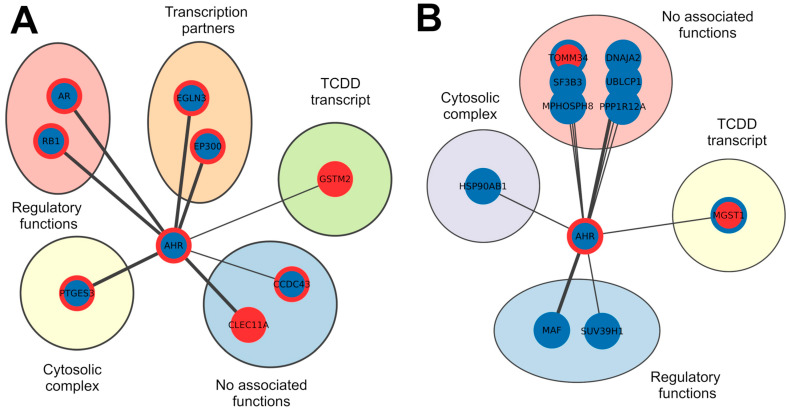
Networks of AHR interactors that have significantly worse survival from the Kaplan–Meier analysis. Panel (**A**) groups proteins upregulated after exposition to TCDD, while downregulated nodes after exposition to TCDD are reported in panel (**B**). Red borders mark upregulated nodes, while blue is for those that were downregulated. Fulfilled red or blue nodes are used to highlight up- or downregulated nodes in both TCDD and ccRCC samples. The thickness of the lines represents the number of sources reporting the interaction.

To gain a deeper understanding of the involvement of these proteins in the context of ccRCC, we employed a Kaplan–Meier plotter to assess the correlation between the expression of 52 proteins (22 upregulated and 30 downregulated in TCDD) (Table 2 and Figure 3A,B) and the survival rate of patients with this tumour type. We found that 16 upregulated and 23 downregulated proteins are significantly associated with worse survival rates, as indicated by their expression levels (*p*-value FDR < 0.05). To identify in our dataset which interactors are responsible for the correlation, we compared the expression levels of 39 proteins—correlated with poorer survival—with the expression levels found in the ccRCC GEO datasets. There were nine upregulated and ten downregulated proteins, whose expression levels consistently align with a worse survival rate (Table 3 and Figure 4), as determined by the Kaplan–Meier analysis.

## 4. Discussion

The recognition of the ARNT as the transcriptional binding partner shared between the AHR and HIF1A highlights the intricate interplay and regulation within these pathways involved in environmental signal responses. The modulation of the AHR has diverse effects on cancer cell behaviour in a cell-specific manner, influencing cell proliferation, invasion, and differentiation [26]. The deletion of the AHR in different cancer types has been associated with alterations in cell proliferation, invasion, and differentiation [20]. The intricate interplay between the AHR and signalling pathways, such as TGF-β, PI3K/AKT/mTOR, NF-κB, FAK/c-Src, and Wnt5a/b-β-catenin, further complicates its role in cancer development [20]. The ligand-activated AHR is reported to either inhibit or induce specific signalling pathways, thereby influencing cancer cell functions [19,20,21,22,23,24,25,26], making it a potential target for cancer-specific therapies as it was evaluated in breast cancer, hepatocellular carcinoma, and melanoma; however, a comprehensive understanding of these contrasting functions is still to be explained. In this work, we investigated the canonical response to TCDD mediated by the aryl hydrocarbon receptor (AHR) and the expression of its interactors, comparing exposure to the receptor’s most potent ligand with the ccRCC pathology condition that precludes the main interaction with the ARNT/HIF1B. Our hypothesis is that sustained competition between the AHR and HIF1A for binding to the ARNT/HIF1B during prolonged exposure to TCDD may lead to significant deregulation of key cellular pathways. Using Cytoscape, we constructed a network centred on the AHR to visualize GEO datasets and identify differentially expressed interactors in the two conditions. Our analysis yielded 36 upregulated and 50 downregulated interactors after TCDD exposure, with 96 interactors exhibiting undefined expression. Further examination of the upregulated and downregulated sets revealed distinct expression patterns in the tumour context. We identified four clusters based on expression levels in the two conditions, classifying interactors into groups such as “Cytosolic complex”, “Regulatory functions”, “Transcription partners”, “TCDD transcript”, “Degradation (no TCDD)”, and “No function”. These data were then correlated with patient survival analysis on ccRCC using a Kaplan–Meier plotter. From the 50 investigated proteins, 9 and 10 proteins from the upregulated and downregulated sets, respectively, were associated with worse survival rates in ccRCC patients. Additionally, two proteins—the AR and RB1—were upregulated by TCDD exposure and downregulated in ccRCC, worsening patient survival rates [62]. The ambiguous role of hormone receptors, particularly the AR, has been extensively studied, revealing its involvement in metastatic migration/invasion processes and its differential regulation of VEGF-A vs. VEGF-C under different oxygen conditions in ccRCC cells [12,20,63,64,65,66,67]. RB1, a tumour suppressor, plays a crucial role in regulating the G1/S transition of the cell cycle [67]. In ccRCC, RB1 often undergoes copy number alterations, impacting cell cycle progression [42,68]. TCDD-induced G1 cell cycle arrest involves a reduction in phosphorylated RB1, facilitated by the direct interaction between the AHR and RB1, protecting RB1 from CDK2/4-mediated phosphorylation [19,69,70]. Our findings suggest that targeting the AHR could hold therapeutic potential in ccRCC; the activation of this receptor, despite the constitutive HIF1A triggering, is shown to potentiate the tumour suppressor behaviour of both the AR and RB1. In summary, this study provides insights into the condition of TCDD-activated AHR interactors within the context of ccRCC. Utilizing computational approaches and survival analysis, we identified potential therapeutic approaches, specifically AR and RB1 enhancement. Ligands of the aryl hydrocarbon receptor (AhR) are categorized into groups such as xenobiotic, endobiotic, and related compounds. It was proposed that ligands within each category may share similar functional activities, differing primarily in their relative potency. Alternatively, these ligands can be viewed as selective modulators (SAhRM), where different SAhRM groups may exhibit overlapping functions, but their genomic and biological activities can vary [71]. Previous investigations in the literature explored this concept, showing that these modulators can have specific agonist and antagonist effects on various cells and tissues. For instance, alkyl polychlorinated dibenzofurans (PCDFs) with alternative substitutions (1,3,6,8- and 2,4,6,8-) and substituted diindolylmethanes (DIMs) can bind to the AHR, leading to inhibitory interactions between the AHR and the estrogen receptor (ER). This interaction mirrors the effects observed with TCDD, including the suppression of mammary tumour growth [72]. The strategic activation of the AHR through a selective AHR modulator (SAhRM) may offer effective anti-tumour therapy in VHL-mutated ccRCC, reducing the need for surgical interventions [71,73].

## Figures and Tables

**Table 1:** Derivation of nodes and edges for each network.

	BIOGRID	HIPPIE	STRING	IntAct	KEGG	Merged
**Nodes**	126	122	20	14	50	182
**Edges**	150	122	52	17	49	327

**Table 2:** List of proteins included in the PPIN, with expression levels found upon TCDD exposition and in ccRCC cells.

Node Name	Expression Levels in at Least Three out of the Five Datasets		Kaplan–Meier Analysis		Expression Level Validation with the ccRCC GEO Datasets
Protein ID	TCDD	ccRCC	Overall Survival (*p*-Value FDR)	LOW Surv. Expression Level	
AHR	HIGH	LOW	$5.47\times 10^{-4}$	LOW	X
ANAPC16	LOW	HIGH	$2.89\times 10^{-4}$	LOW	
AR	HIGH	LOW	$6.24\times 10^{-11}$	LOW	X
ARNT2	HIGH	HIGH	$2.60\times 10^{-2}$	LOW	
BRCA1	HIGH	LOW	$7.22\times 10^{-2}$	LOW	
CCDC43	HIGH	LOW	$9.01\times 10^{-5}$	LOW	X
CHMP1A	LOW	LOW	$7.22\times 10^{-2}$	HIGH	
CLEC11A	HIGH	HIGH	$5.20\times 10^{-3}$	HIGH	X
CYP1B1	LOW	LOW	$1.13\times 10^{-3}$	HIGH	
DDB1	LOW	HIGH	$6.76\times 10^{-7}$	LOW	
DNAJA2	LOW	LOW	$1.95\times 10^{-10}$	LOW	X
EGLN3	HIGH	LOW	$1.38\times 10^{-2}$	LOW	X
EP300	HIGH	LOW	$2.13\times 10^{-5}$	LOW	X
EPHX2	LOW	HIGH	$2.13\times 10^{-5}$	LOW	
EPHX4	HIGH	LOW	$9.98\times 10^{-2}$	HIGH	
FASN	LOW	LOW	$1.10\times 10^{-5}$	HIGH	
GSTA4	LOW	HIGH	$3.12\times 10^{-3}$	LOW	
GSTM2	HIGH	HIGH	$4.70\times 10^{-3}$	HIGH	X
GSTM3	LOW	HIGH	$1.13\times 10^{-3}$	LOW	
GSTM4	LOW	HIGH	$1.51\times 10^{-1}$	LOW	
GSTM5	HIGH	HIGH	$1.71\times 10^{-1}$	LOW	
HSP90AB1	LOW	LOW	$2.24\times 10^{-4}$	LOW	X
HSPB8	HIGH	LOW	$7.81\times 10^{-2}$	LOW	
IVNS1ABP	HIGH	HIGH	$9.78\times 10^{-7}$	LOW	
KIF14	HIGH	LOW	$1.82\times 10^{-6}$	HIGH	
MAF	LOW	LOW	$7.43\times 10^{-4}$	LOW	X
MGST1	LOW	HIGH	$3.39\times 10^{-2}$	HIGH	X
MGST2	LOW	HIGH	$2.45\times 10^{-6}$	LOW	
MPHOSPH8	LOW	LOW	$2.50\times 10^{-3}$	LOW	X
NCOA1	HIGH	HIGH	$2.69\times 10^{-3}$	LOW	
NCOA7	LOW	HIGH	$9.78\times 10^{-7}$	LOW	
NR2F1	LOW	HIGH	$2.69\times 10^{-3}$	LOW	
PARP1	LOW	LOW	$5.02\times 10^{-2}$	HIGH	
PPP1R12A	LOW	LOW	$3.24\times 10^{-2}$	LOW	X
PSMC2	LOW	LOW	$1.09\times 10^{-1}$	HIGH	
PSMD3	LOW	LOW	$4.00\times 10^{-5}$	HIGH	
PTGES3	HIGH	LOW	$2.89\times 10^{-4}$	LOW	X
PUS7	LOW	LOW	$8.77\times 10^{-2}$	HIGH	
RAF1	HIGH	LOW	$1.30\times 10^{-1}$	HIGH	
RB1	HIGH	LOW	$2.60\times 10^{-6}$	LOW	X
RBX1	LOW	LOW	$2.50\times 10^{-3}$	HIGH	
RELA	LOW	LOW	$9.98\times 10^{-2}$	HIGH	
SF3B3	LOW	LOW	$7.43\times 10^{-4}$	LOW	X
SMAD4	LOW	HIGH	$2.64\times 10^{-5}$	LOW	
SUMO2	HIGH	LOW	$1.57\times 10^{-2}$	HIGH	
SUV39H1	LOW	LOW	$2.50\times 10^{-3}$	LOW	X
TERF2IP	LOW	HIGH	$1.71\times 10^{-1}$	HIGH	X
TOMM34	LOW	HIGH	$7.06\times 10^{-5}$	HIGH	X
TRIM52	HIGH	LOW	$9.77\times 10^{-3}$	HIGH	
UBLCP1	LOW	LOW	$2.08\times 10^{-2}$	LOW	X
UGT1A1	HIGH	HIGH	$1.71\times 10^{-1}$	HIGH	
VIM	HIGH	LOW	$2.50\times 10^{-3}$	HIGH	

**Table 3:** Summary tables of the nodes that have significantly worse survival in ccRCC.

Upregulated Nodes (TCDD 100 nM for 6 h)				Downregulated Nodes (TCDD 100 nM for 6 h)			
Gene ID	Expression Levels in ccRCC	Overall Survival (*p*-Value FDR)	Expression Levels Correlated to Worse Survival Rate	Gene ID	Expression Levels in ccRCC	Overall Survival (*p*-Value FDR)	Expression Levels Correlated to Worse Survival Rate
CCDC43	**LOW**	$9.01\times 10^{-5}$	**LOW**	DNAJA2	**LOW**	$1.95\times 10^{-10}$	**LOW**
AR	**LOW**	$6.24\times 10^{-11}$	**LOW**	HSP90AB1	**LOW**	$2.24\times 10^{-4}$	**LOW**
EP300	**LOW**	$2.13\times 10^{-5}$	**LOW**	MAF	**LOW**	$7.43\times 10^{-4}$	**LOW**
RB1	**LOW**	$2.60\times 10^{-6}$	**LOW**	MGST1	**HIGH**	$3.39\times 10^{-2}$	**HIGH**
EGLN3	**LOW**	$1.38\times 10^{-2}$	**LOW**	MPHOSPH8	**LOW**	$2.50\times 10^{-3}$	**LOW**
GSTM2	**HIGH**	$4.70\times 10^{-3}$	**HIGH**	PPP1R12A	**LOW**	$3.24\times 10^{-2}$	**LOW**
AHR	**LOW**	$5.47\times 10^{-4}$	**LOW**	SF3B3	**LOW**	$7.43\times 10^{-4}$	**LOW**
CLEC11A	**HIGH**	$5.20\times 10^{-3}$	**HIGH**	SUV39H1	**LOW**	$2.50\times 10^{-3}$	**LOW**
PTGES3	**LOW**	$2.89\times 10^{-4}$	**LOW**	TOMM34	**HIGH**	$7.06\times 10^{-5}$	**HIGH**
				UBLCP1	**LOW**	$2.08\times 10^{-2}$	**LOW**

## Data Availability

The datasets generated during and/or analyzed during the current study are available from the corresponding author upon reasonable request.

## Abbreviations

VHL, pVHL	von Hippel–Lindau tumour suppressor
HIFs	Hypoxia-inducible factors
ccRCC	Clear cell renal cell carcinoma
AHR	Aryl hydrocarbon receptor
HIF-1β, ARNT	Aryl hydrocarbon receptor nuclear translocator
TCDD	Dioxin, 2,3,7,8-tetrachlorodibenzo-p-dioxin
AR	Androgen receptor
RB1	Retinoblastoma-associated protein
SAhRMs	Selective AHR modulators
bHLH-PAS	Basic helix–loop–helix-PER-ARNT-SIM transcription factors
pNETs	Non-functioning pancreatic neuroendocrine tumours
RCC	Renal cell carcinoma
VCB complex	Complex formed by pVHL, elongin-B, elongin-C, and Cullin-2
ELOB	Elongin-B
ELOC	Elongin-C
CUL2	Cullin-2
HIF-1α	Hypoxia-inducible factor 1-α
HSP90	Heat shock protein 90
p23	Prostaglandin E synthase 3
XAP2	AH receptor-interacting protein
XREs	Xenobiotic-responsive elements
DREs	Dioxin-responsive elements
Cyp1a	Cytochrome P450, family 1, subfamily A, polypeptide 1
Cyp1b	Cytochrome P450, family 1, subfamily B, polypeptide 1
GSTA1	Glutathione S-transferase A1
EPHX1	Epoxide hydrolase 1
PAI2	Plasminogen activator inhibitor 2
p300	Histone acetyltransferase p300
CREBBP	CREB-binding protein
NCOA1/2/3	Nuclear receptor coactivator 1/2/3
NRIP1	Nuclear receptor-interacting protein 1
ER	Estrogen receptor
HepaRG	Human hepatic in vitro line
MCF7	Human breast cancer cell line (Michigan Cancer Foundation-7)
HepG2	Human liver cancer cell line
786-O	Human renal cancer cell line
EGA	European Genome–Phenome Archive
TCGA	The Cancer Genome Atlas
GEO	Gene Expression Omnibus
HR	Hazard ratio
PPI	Protein–protein interaction
DEGs	Differentially expressed genes
GSTA5	Glutathione S-transferase A5
GSTM2	Glutathione S-transferase Mu 2
UGT2B11	UDP-glucuronosyltransferase 2B11
PPIN	Protein–protein interaction network
VEGF-A/C	Vascular endothelial growth factor A/C
CDK2/4	Cyclin-dependent kinase 2/4
